# Supplementary material for: Effect of the Fat Eaten at Breakfast on Lipid Metabolism: A Crossover Trial in Women with Cardiovascular Risk
Source: Nutrients. 2020 Jun 6;12(6):1695. doi: 10.3390/nu12061695 (PMC7352537; doi:10.3390/nu12061695)
Supplement: Supplementary file 1 [file nutrients-12-01695-s001.pdf]

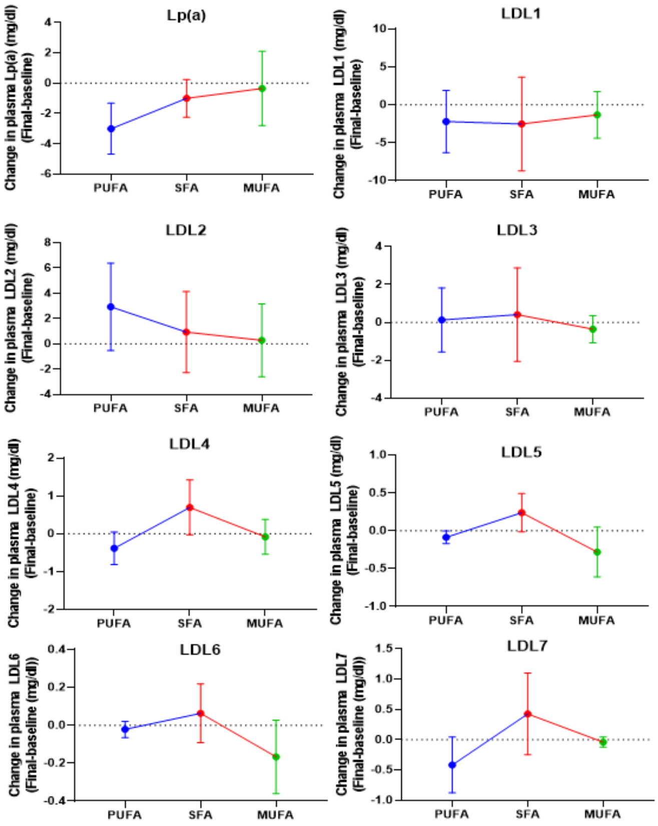

Figure S1. Changes in Lp(a) and LDL subfractions. Data represent estimated treatment differences (final- baseline) values and 95% CI. Data derive from those women who completed the study (n = 53). * represents differences within the same group (final- baseline). *$p < 0.050$, ** $p < 0.010$, *** $p < 0.001$. Within-groups differences are indicated as p_{group}. Further information is available at Table S2.

Table S1. Fatty acid composition of the different types of breakfast.

	MARGARINE (60g fat/100g)	BUTTER (81g fat/100g)	VIRGIN OLIVE OIL (99g fat/100g)
**SFA**	12.8	51.4	14.5
Butyric acid (4:0)	-	3.2	-
Caproic acid (6:0)	-	2.0	-
Caprylic acid (8:0)	0.1	1.2	-
Capric acid (10:0)	0.1	2.5	-
Lauric acid (12:0)	1.7	2.6	-
Myristic acid (14:0)	0.6	7.4	-
Palmitic acid (16:0)	5.6	21.7	14.4
Stearic acid (18:0)	4.2	9.9	0.1
**MUFA**	15.3	21.0	71
Palmitoleic acid (16:1n-9)	-	1.1	-
Oleic acid (18:1n-9)	15.3	19.9	71
**PUFA**	29.2	3.0	10
Linoleic acid(18:2n-6)	28.9	2.7	9.1
α-linolenic acid (18:3n-3)	0.3	0.3	0.9
Cholesterol (mg)	-	215	-

Data represents g fatty acid/100 g food) excepting cholesterol, which indicates mg/100g food. SFA: saturated fatty acids; MUFA: monounsaturated fatty acids; PUFA: polyunsaturated fatty acids.

Table S2. Effect of 30-day intervention with the different breakfasts on plasma lipoproteins and their subfractions.

	**PUFA pre**	**PUFA post**	**Change (*p*)**	**SFA pre**	**SFA post**	**Change (*p*)**	**MUFA pre**	**MUFA post**	**Change (*p*)**	$p_{\Delta group}$
**Lp(a)**	**24.4 ± 22.6**	**21.4 ± 20.1**	**-3.0 (0.001)**	23.1 ± 19.9	22.2 ± 20.5	-0.9 (0.084)	20.6 ± 18.0	20.1 ± 16.3	-0.5 (0.683)	**0.013**
**VLDL**	**29.5 ± 9.9**	**24.8 ± 7.7**	**-4.8 (0.001)**	**22.7 ± 7.7**	**19.7 ± 7.1**	**-3.0 (0.011)**	24.8 ± 9.5	23.8 ± 9.4	-1.0 (0.092)	**0.036**

**Total IDL**	**48.5 ± 11.7**	**44.2 ± 10.9**	**-4.3 (0.006)**	**39.8 ± 8.6**	**46.3 ± 11.1**	**6.5 (<0.001)**	46.0 ± 10.1	45.1 ± 7.4	-0.9 (0.440)	0.071
**IDL-a**	13.8 ± 5.4	13.3 ± 6.1	-0.5 (0.576)	13.6 ± 5.5	12.6 ± 5.4	-1.0 (0.368)	13.0 ± 4.6	12.7 ± 3.8	-0.3 (0.570)	0.511
**IDL-b**	12.8 ± 3.9	13.1 ± 4.3	0.4 (0.494)	**12.6 ± 3.9**	**14.3 ± 4.1**	**1.7 (0.006)**	12.9 ± 3.8	12.4 ± 3.2	-0.4 (0.368)	0.358
**IDL-c**	**22.0 ± 6.7**	**17.8 ± 4.8**	**-4.1 (0.001)**	**13.7 ± 4.4**	**19.5 ± 6.1**	**5.8 (-0.001)**	20.2 ± 5.8	20.0 ± 4.1	-0.2 (0.815)	0.056
**Total LDL**	67.8 ± 13.8	67.9 ± 16.9	0.1 (0.963)	63.1 ± 15.2	63.4 ± 14.5	0.3 (0.887)	67.9 ± 19.2	65.9 ± 16.7	-2.0 (0.370)	0.227
**LDL1**	33.8 ± 9.0	31.6 ± 12.1	-2.2 (0.295)	34.0 ± 15.0	30.9 ± 12.0	-1.4 (0.311)	35.0 ± 12.9	33.6 ± 9.3	-1.3 (0.376)	0.925
**LDL2**	26.8 ± 8.7	29.7 ± 10.5	2.9 (0.101)	23.7 ± 10.6	24.6 ± 10.4	0.9 (0.538)	25.4 ± 12.4	25.7 ± 10.1	0.3 (0.831)	0.403
**LDL3**	5.8 ± 4.7	6.0 ± 5.4	0.2 (0.876)	5.8 ± 6.3	6.3 ± 7.3	0.4 (0.729)	6.0 ± 5.8	5.6 ± 6.1	-0.4 (0.312)	0.498
**LDL4**	1.0 ± 1.5	0.6 ± 1.2	-0.4 (0.066)	0.5 ± 1.0	1.2 ± 2.4	0.7 (0.060)	0.9 ± 1.7	0.8 ± 1.3	-0.1 (0.712)	0.610
**LDL5**	**0.1 ± 0.3**	**0.0 ± 0.0**	**-0.1 (0.050)**	0.0 ± 0.3	0.3 ± 0.8	0.3 (0.058)	0.3 ± 0.8	0.0 ± 0.0	-0.3 (0.092)	0.318
**LDL6**	0.0 ± 0.1	0.0 ± 0.0	-0.0 (0.317)	0.0 ± 0.2	0.1 ± 0.5	0.1 (0.400)	0.3 ± 0.9	0.1 ± 0.4	-0.2 (0.172)	0.700
**LDL7**	0.2 ± 1.0	0.0 ± 0.0	-0.1 (0.081)	0.1 ± 0.3	0.5 ± 2.3	0.3 (0.205)	0.1 ± 0.4	0.0 ± 0.2	-0.1 (0.344)	0.440
**HDL**	**58.5 ± 14.2**	**71.1 ± 14.1**	**12.6 (<0.001)**	**69.8 ± 11.6**	**64.8 ± 12.0**	**-5.0 (0.002)**	**59.6 ± 15.7**	**64.2 ± 15.5**	**4.6 (0.017)**	**0.010**

Data represent mean ± sd. Change data refer to the plasma value of every variable compared to the baseline values and statistical significance was indicated in parenthesis. Repeated-measures ANCOVA was employed to evaluate possible statistical differences, using age and BMI as covariates. To assess differences in the treatment effect, a new RM-ANCOVA ($p_{\Delta group}$) was performed comparing the changes produced at the end of treatment with respect to the baseline values in each group, also using age and BMI as covariates.

Table S3. Effect of 30-day intervention with the different breakfasts on plasma lipoproteins attending to the electrophoretic mobility and plasma apolipoproteins.

	PUFA pre	PUFA post	Change	SFA pre	SFA post	Change	MUFA pre	MUFA post	Change	$p_{\Delta group}$
**Chilomicrons**	**1.0 ± 0.8**	**0.7 ± 0.5**	**-0.3 (0.021)**	0.7 ± 0.5	0.7 ± 0.4	0.0 (0.529)	**0.9 ± 0.5**	**0.7 ± 0.4**	**-0.2 (0.003)**	0.518
**β-lipoprotein**	**49.9 ± 7.0**	**45.0 ± 4.6**	**-4.9 (<0.001)**	**49.5 ± 5.9**	**52.8 ± 7.3**	**3.3 (0.025)**	**44.6 ± 3.5**	**47.3 ± 4.4**	**2.7 (<0.001)**	0.671
**Preβ-lipoprotein**	**15.0 ± 5.8**	**17.1 ± 5.5**	**2.1 (0.017)**	**17.0 ± 6.8**	**12.5 ± 5.1**	**-4.5 (<0.001)**	15.5 ± 6.3	14.7 ± 4.7	-0.8 (0.292)	0.384
**α-lipoprotein**	**34.6 ± 8.1**	**37.2 ± 5.7**	**2.6 (0.018)**	32.8 ± 8.8	34.0 ± 8.3	1.2 (0.399)	**38.9 ± 5.9**	**37.1 ± 6.5**	**-1.8 (0.007)**	0.359
**APO-AI**	**165.5 ± 17.1**	**181.8 ± 20.4**	**16.3 (<0.001)**	**181.6 ± 18.3**	**176.1 ± 18.9**	**-5.5 (0.004)**	178.7 ± 19.2	177.0 ± 18.9	-1.7 (0.258)	0.281
**APO-AII**	34.8 ± 4.9	35.1 ± 4.7	0.3 (0.334)	**35.3 ± 5.0**	**33.3 ± 4.6**	**-2.0 (<0.001)**	**34.3 ± 4.3**	**32.0 ± 6.1**	**-2.3 (0.001)**	**0.026**
**APO-B**	**87.1 ± 18.2**	**83.0 ± 14.4**	**-4.1 (0.018)**	86.3 ± 17.1	87.8 ± 20.0	1.5 (0.359)	94.7 ± 18.4	95.1 ± 18.9	0.4 (0.828)	0.431
**APO-E**	**4.4 ± 0.9**	**4.7 ± 1.1**	**0.3 (<0.001)**	4.6 ± 0.9	4.4 ± 1.0	-0.2 (0.128)	4.4 ± 1.0	4.4 ± 1.0	0.0 (0.745)	0.399

Data represent mean ± sd. Change data refer to the plasma value of every variable compared to the baseline values and statistical significance was indicated in parenthesis. Repeated-measures ANCOVA was employed to evaluate possible statistical differences, using age and BMI as covariates. To assess differences in the treatment effect, a new RM-ANCOVA ($p_{\Delta group}$) was performed comparing the changes produced at the end of treatment with respect to the baseline values in each group, also using age and BMI as covariates.
